# Face Culture and Prosocial Value Conflict: A Developmental Investigation of Children’s White Lie Decisions Between Emotional Comfort and Long-Term Goals

**DOI:** 10.3390/bs16040593

**Published:** 2026-04-15

**Authors:** Yunrui Sun, Zhijie Du, Jinhai Cui

**Affiliations:** 1 Faculty of Education, Tianjin Normal University, Tianjin 300380, China; 13693128863@163.com; 2 School of Politics and Administration, Tianjin Normal University, Tianjin 300380, China; jinhai1917@163.com

**Keywords:** prosocial values, white lie, moral decision-making, children’s development, face culture, value conflict

## Abstract

White lie-telling reflects children’s integration of moral cognition and situational adaptation, yet its mechanisms in prosocial dilemmas remain understudied in Chinese cultural contexts that prioritize “face-saving”—a core construct that shapes interpersonal behavior in Eastern societies. This study investigates how situational cues and developmental differences shape children’s white lie decisions by disentangling the interactive effects of external expectations and recipient presence. A total of 629 children aged 4–11 years (Study 1) and 6–11 years (Study 2) participated in two studies using a modified “painting evaluation task” Study 1 manipulated emotional expectation and recipient presence to establish baseline situational effects, while Study 2 introduced target expectation to create a prosocial value conflict between providing immediate emotional comfort and supporting long-term developmental goals. The Study 1 showed the highest white lie rate under the “emotional expectation + recipient presence” condition, with white lie rates exhibiting a significant developmental increase with age. Binary logistic regression identified these two factors as critical predictors of children’s white lie behavior. In Study 2, amid such prosocial value conflicts, older children showed lower white lie rates than younger peers, who prioritized others’ long-term goals via cost benefit analysis. Notably, recipient presence still moderated face-saving decisions, even for older children. This research makes three key contributions to the field. Firstly, it integrates Chinese “face culture” into situational manipulation, highlighting recipient presence as a culture-specific moderator and mitigating the Western-centric bias in prior research. Secondly, it constructs a prosocial moral dilemma to uncover children’s developmental transition from emotion-driven to value-based rational decision-making, extending existing developmental theories on moral cognition. Thirdly, it advances understanding of prosocial lying motivation beyond blind empathy by quantifying the interactive effects of dual expectations and revealing that children engage in deliberate cost benefit analysis that aligns with others’ overall long-term interests.

## 1. Introduction

### 1.1. Situational Factors, Face Culture, and Children’s White Lies

Children are socialized from an early age and encouraged to be honest in most social environments. However, they are also taught, either explicitly or implicitly, not to tell the truth if it might hurt others in certain social situations (Liang et al., 2025). Therefore, children tell white lies to be polite or to protect the listener’s feelings. White lie-telling is an indispensable and mature social behavior in the course of children’s social development (Heyman et al., 2020; Talwar et al., 2016). Hence, the study of children’s white lies is helpful to understand how children use complicated social cognition to achieve more positive and friendly social relations.

Relevant studies indicate that the situation is a key factor affecting children’s white lie behavior (Heyman et al., 2020; Potnikowska & Filip, 2024; Sun et al., 2022b; Talwar et al., 2007). Fu and Lee (2007) found that children tell more white lies to familiar than unfamiliar adults and display more flattery when the other party is present. Arruda and Souza (2020) discovered that children aged 7–11 years deemed prosocial lying to safeguard their friends as more justifiable than lying to protect their own or group interests. This finding implies that children are sensitive to circumstances and can modify their verbal and non-verbal behaviors in accordance with situational elements. Although situational factors are of critical significance for children’s white lie behavior, prior studies have predominantly centered on Western samples. There are scarce cross-cultural investigations on white lie behavior (Liu, 2017; Talwar & Lee, 2002; Talwar et al., 2007). Situational factors are intricate, and the presence or absence of the other party/recipient constitutes the most crucial situational factor in China. The underlying cause is face culture, which assumes a complex and indispensable role in Chinese social life. It emphasizes that within the social milieu, it is requisite to safeguard the emotions of others and to conceal our true sentiments and views of others. Consequently, within the context of Chinese face culture, children are cognizant that protecting another’s face—or saving face—is an essential aspect of interpersonal communication when the other party is present. Research on Chinese face culture has identified its unique cultural connotation in shaping social behavior, which is distinct from Western social norm research and has become a key direction of cross-cultural moral development research (Liu, 2017; Fu & Lee, 2007). While white lies are observed across cultures, the motivations underlying them may differ substantially. In Chinese culture, “face” plays a unique role in moral socialization. It emphasizes interpersonal harmony, the protection of others’ social standing, and the avoidance of public embarrassment (Liu, 2017). Children are socialized from an early age to prioritize these relational goals over individual honesty, especially when the other person is present. In contrast, Western individualistic cultures tend to value personal authenticity and directness, even at the risk of causing temporary discomfort (Heyman et al., 2020). However, this does not imply that Western children tell fewer white lies; in fact, research shows high rates of prosocial lying in Western samples as well (Talwar & Lee, 2002; Warneken & Orlins, 2015). This suggests that while face culture may amplify the salience of recipient presence, there are also universal social norms (e.g., politeness and empathy) that promote white lie-telling across cultures. Therefore, the key cultural difference may lie not in the frequency of white lies, but in the relative weight children assign to different prosocial values—emotional comfort vs. long-term benefit—when these values conflict. Future cross-cultural research should directly compare Chinese and Western children’s responses to the moral dilemmas introduced in Study 2. Notably, the cultural particularity of Chinese face culture in the moral socialization context lies in its other-oriented face maintenance logic and situation-dependent interpersonal harmony norms (Liu, 2017; Fu & Lee, 2007), which is essentially different from the Western face concept centered on individual self-presentation and impression management (Lim et al., 2020). This cultural classification difference means that Chinese children’s prosocial decision-making is more sensitive to the presence of others and the maintenance of on-site interpersonal relationships, while Western children’s relevant behaviors are more guided by individual moral principles and universal politeness norms.

Thus, we hypothesized that children tell more white lies in the presence of the person than in their absence (Hypothesis 1). The occurrence of white lie behavior is based on the speaker’s consideration that telling the truth will cause negative feelings for the listener, while lying can improve the listener’s experience (Ong & Nilsen, 2025). Thus, for the benefit of the listener, white lie-tellers not only understand others’ feelings but are also motivated to alleviate the pain of others or to help them (Casey et al., 2020; Erat & Gneezy, 2012; Popliger et al., 2011). Studies have shown that when children received a disappointing gift from an experimenter, they did not express their dislike of the gift when the experimenter was present. However, they expressed their disappointment to another experimenter in the absence of the gift-giver (Hudson & Jacques, 2014; Talwar et al., 2007). In Talwar and study, the experimenter asked children how they looked when red dots appeared on the experimenter’s nose before taking photographs. The results indicate that even 3-year-old children could be considerate of another person’s feelings and hide the fact that the experimenter did not look good. These studies show that children will make every effort to avoid causing negative emotions for another and will cater to their emotional expectations. Therefore, we hypothesized that children are more likely to tell white lies in situations where the recipient has emotional expectations, compared with those where they do not (Hypothesis 2).

### 1.2. From Emotional Drive to Cost Benefit Analysis: A Developmental Shift

Children are inclined to give positive comments to others. They have good intentions, but there are gray areas. Telling white lies blindly to make others feel better does not necessarily produce satisfactory results (Lupoli et al., 2017). Studies show that children do not necessarily make prosocial decisions blindly. The concept of cost benefit analysis has been proposed—individuals make mature decisions in various environments (Kool et al., 2010; Talwar et al., 2017). The cost benefit calculation method is the basis for children’s inference and evaluation. When individuals make moral judgments, they also conduct a cost benefit analysis for others. Decisions about whether to tell the truth are influenced by the likelihood that the information will be helpful to the recipient. Heyman et al. (2020) adapted the reverse rouge paradigm to study children; the experimenter—with a prominent red mark on her nose—asked children whether she looked okay before having their picture taken. Although honesty might cause emotional distress for the recipient, when children found that telling the truth was helpful to the experimenter, most children chose to be honest. This reveals that children conduct a cost benefit analysis for others before telling a white lie. Faced by the moral dilemma arising from the conflict between the two prosocial values—the white lie can mollify another’s current emotions, whereas honesty may also provide benefits for their long-term development. However, preschool children, who are predominantly in the pre-moral stage and whose thinking development is self-centered and susceptible to emotion, are incapable of effectively judging the morality of white lies. As they progress in age, children possess increasingly flexible and rational moral judgments regarding white lies. Moreover, they can render judgments based on the diverse consequences that white lies bring about for themselves and others (Cui et al., 2026). In prosocial moral decision-making, children endeavor to maximize the overall target interests of others. Consequently, we hypothesized that when a conflict between two interests is perceived, children will abandon short-term emotional interests and contemplate the long-term interests of others (Hypothesis 3).To systematically investigate developmental differences, we propose the following Hypothesis 3: Older children (9–11 years) are more likely than younger children (4–5 years) to engage in cost benefit analysis and make rational white-lie decisions when faced with prosocial value conflicts. We also introduce key cognitive constructs underlying white lie development: theory of mind (attributing mental states to others), executive function (goal-directed cognitive processes), and empathy (understanding and sharing others’ emotions). These abilities develop with age and are critical for appropriate white lie-telling (Sun et al., 2022a; Lv & Sun, 2022). Additionally, we extend the discussion of face culture by comparing it with Western norms. In Chinese culture, “saving face” emphasizes interpersonal harmony and avoiding public embarrassment. In contrast, Western cultures may prioritize individual authenticity and directness, even at the risk of temporary discomfort (Liu, 2017). This cultural distinction is crucial for understanding how children across societies navigate prosocial dilemmas involving white lies.

### 1.3. The Present Study: Research Questions, Design, and the Logical Link Between Two Studies

To investigate how situational factors and developmental changes shape children’s white lie decisions, we adopted and modified the “painting evaluation task paradigm” utilized in Fu and Lee (2007). Rather than rating the paintings on a continuous scale, a binary forced selection approach was implemented to explore whether children tell white lies. Hence, the coding principle of this study was that when children evaluated a poor target painting as “good” it was coded as a “white lie” Conversely, when a poor target painting was evaluated as “bad” it was coded as “truth”.

We conducted two experiments. Study 1 aimed to establish the basic effects of situational factors (emotional expectation and the recipient’s presence/absence) on children’s white lies across a wide age range (4–11 years). In Study 1, children were randomly divided into four groups based on the presence or absence of the artist and the presence or absence of the artist’s emotional expectation. This allowed us to test Hypotheses 1 and 2 regarding the independent and interactive effects of these factors.

Building on the findings of Study 1, Study 2 was designed to create a more complex moral dilemma that would test Hypothesis 3. Specifically, Study 2 introduced a second, competing prosocial cue: the artist’s target expectation. This created a conflict between two prosocial values: providing immediate emotional comfort versus supporting the recipient’s long-term goals. Given the cognitive demands of this cost benefit analysis, we focused on primary school children (6–11 years). We investigated whether, when faced with this value conflict, children would undertake a cost benefit analysis and ultimately make a rational white lie decision that prioritizes the long-term interests of others.

Individuals have differences in their ability to empathize. In addition, some studies have found that individuals with the trait of positive empathy have a higher likelihood of understanding the psychological state of another, actively resonating with that person, and producing more prosocial behaviors (Nagar et al., 2020; Kline et al., 2019; Yue et al., 2021). Other studies also found that children with higher scores of affinity traits, including compassion and altruism, are more likely to help others by lying (Demedardi et al., 2021; Sun et al., 2022a). Hence, we measured the empathy ability of each participant before the experiment to eliminate the impact of trait empathy on the experimental results. Therefore, the children’s empathy ability is the control variable in the study.

## 2. Study 1

### 2.1. Participants

A total of 400 children between 4 and 11 years of age (Mage = 89.88 months, SD = 3.60, 52.5% boys) participated in the study. A power analysis (G Power 3.1; Faul et al., 2009) was run to calculate a prior sample size for Study 1, which showed that a sample of 400 participants was sufficient for an effect size (η2p) of 0.1 with significance at the 5% level and power (1-β) of 0.8. The children were predominantly recruited from two kindergartens and two primary schools in a large eastern Chinese city. Stratified random sampling was employed by age (4–5, 6–8, 9–11 years), gender, and socioeconomic background to ensure representative samples and enhance the generalizability of findings.

All of the remaining 393 participants (Mage = 88.4 months, SD = 2.46, 52.7% boys) were initially assigned to one of the experimental conditions, and 7 of them were excluded for failing correctly distinguish between good and bad in the first round of the task. The study was approved by the Research Ethics Committee in the Academy of Psychology and Behavior at Tianjin Normal University, China. All participants gave written informed consent before being enrolled in the experiment, which was obtained from their parents or legal guardians prior to the beginning of the study. For statistical analyses, children were divided into two age groups: young children (4–5 years) and primary school students (6–11 years). However, to provide a more detailed developmental picture, Table 1 presents white lie rates across three age subgroups (4–5, 6–8, and 9–11 years).

The task paradigm of painting evaluation was adopted in this study (Fu & Lee, 2007; Warneken & Orlins, 2015). The study used a 2 (Age: Young children, Primary school students) × 4 (Situational factors: Emotional expectation and Present, Emotional expectation and Absent, No emotional expectation and Present, No emotional expectation and Absent) between-participants design. Participants were randomly assigned to one of the conditions. Emotional expectation: The artist expressed sadness through both verbal cues (“I worked really hard, but it seems I’m not good at it”) and non-verbal cues (frowning, sighing), explicitly conveying the emotional need for positive evaluation. No emotional expectation: The artist only stated “I’ve finished painting” without emotional expression or goal explanation, maintaining a neutral attitude.

### 2.2. Materials and Methods

#### 2.2.1. Empathy Test

In this study, the empathic continuum developed by Stray (Empathy Continuum, EC, Strayer & Roberts, 1997, 2004) was used to test children’s empathic ability. The test contains four emotional stories of happiness, sadness, fear, and anger. Each story describes an emotion. Children’s levels of cognitive and emotional empathy were measured based on their reports on the roles of characters in the story and their own emotional states. The cognitive empathy question was “How does the protagonist feel in the story?”. When the subject had a correct emotional judgment, 1 point was given; otherwise, 0 points were given. The emotional empathy question was “After hearing the story, your feeling is…”. When the subject’s affective response was consistent with the protagonist’s emotion, 1 point was given; otherwise, 0 points were given. The scores for cognitive and emotional empathy ranged from 0 to 4, and the total score for empathy was 0 to 8. Before the test, six graduate students and two advisors were selected to evaluate the emotions evoked by the story. The evaluation results indicate that situational stories can trigger the corresponding emotion, and the consistency coefficient was over 0.85. A total of 18 children evaluated their emotional responses to the story, and the results show that situational stories could trigger the corresponding emotion; the consistency coefficient of the children was over 0.80.

#### 2.2.2. Test Procedure

The classification of motivational cues (emotional vs. target expectation) was based on prior research on prosocial lying (Heyman et al., 2020; Talwar et al., 2017). Emotional expectation was operationalized through verbal and nonverbal expressions of sadness, conveying the need for emotional comfort. Target expectation was operationalized through goal-oriented statements about competition participation, conveying the need for long-term benefit. These two motivational cues were chosen because they represent distinct prosocial values that often conflict in real-world moral dilemmas: providing immediate emotional relief versus facilitating long-term improvement. The key factors influencing this classification include (a) the recipient’s expressed emotional state, (b) the presence or absence of explicit future goals, and (c) the potential trade-off between short-term affect and long-term outcomes. This distinction allowed us to examine how children weigh competing prosocial values when making white lie decisions. To ensure that children perceived the target painting as “bad” only those who correctly distinguished between good and bad paintings in the first round of the task were included in the analysis. Seven children were excluded for failing this criterion.

**Figure 1:**
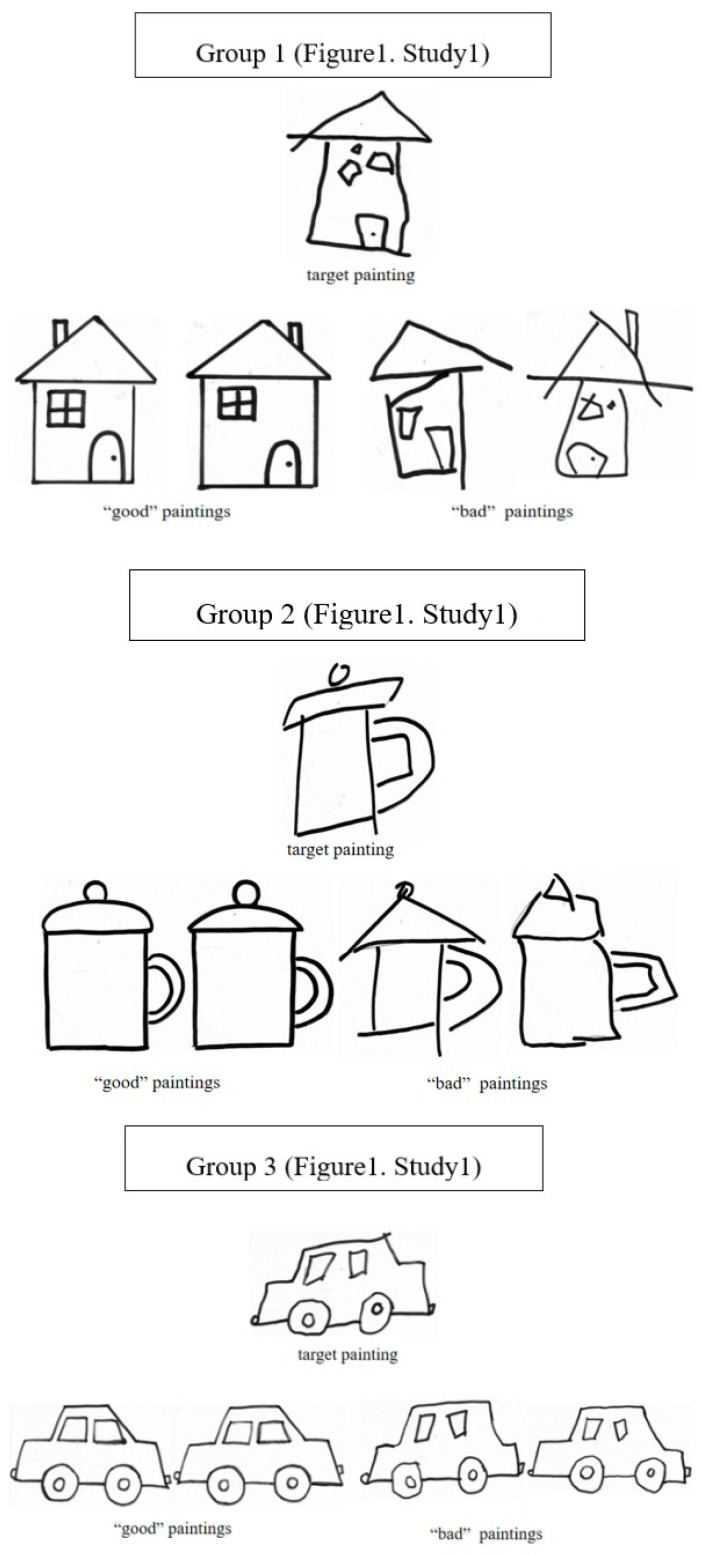
Example image of sample stimuli (Study 1).

The experimental materials were three groups of paintings: houses, cups, and cars (Figure 1). Each group contained five paintings: two “good” paintings with well-coordinated composition and smooth strokes, and three “bad” paintings with poor composition coordination and unsmooth strokes. To ensure children perceived the artist’s effort, the target “bad” painting was presented with visual cues (e.g., sketches with revision traces placed next to it) indicating repeated attempts to improve. To ensure the reliability of the “good” vs. “bad” classification, a pilot study was conducted with 30 children (ages 4–11) who did not participate in the main study. They were asked to classify the paintings into “good-looking” and “bad-looking” categories. The inter-rater agreement was 92%, indicating high reliability (k = 0.89).

Before the experiment, the evaluation criteria for “good” and “bad” paintings were explained to the children. In each experiment, the children were asked to classify the paintings into good-looking (red bag) and bad-looking ones (blue bag). Before experiment, Experimenter 1 (E1) would randomly select one of the “bad” paintings in the third group as the target painting in advance. Each experiment was conducted in two rounds. In the first round, children were asked to classify two good paintings and three bad paintings to the blue and red bags. The second round was conducted after the children’s correct classification. Then E1 asked the children to classify the remaining two “good” and two “bad” paintings in the third group of paintings (one was already taken). Children were invited to the second round of classification only after the first round of correct classification was completed. To avoid the influence of gender advantage or preference on children’s white lie choices, Experimenter 2’s (E2) gender was systematically counterbalanced across all experimental conditions and age groups. In Study 1, E2 was male in 198 sessions (50.4%) and female in 195 sessions (49.6%), with approximately equal distribution across the four conditions (χ^2^ = 1.23, *p* = 0.745). In Study 2, E2 was male in 118 sessions (50.0%) and female in 118 sessions (50.0%), with equal distribution across conditions. All E2s received standardized training on facial expressions, tone of voice, and script delivery, and were kept blind to the experimental hypotheses.

Children were divided into separate groups to perform the following operations. Given the large sample size (N = 393 in Study 1), video recording all sessions was not feasible. Instead, we established coding reliability through pre-testing. Prior to the formal experiment, a pre-test was conducted with 18 children (as described in Section 2.2.1). These sessions were video-recorded and independently coded by two experimenters who were blind to the study hypotheses. Inter-rater agreement was high (k = 0.86), confirming the clarity of the coding criteria. During the formal experiment, children’s responses were recorded in real time by two experimenters independently (placing a painting into the red “good” bag or blue “bad” bag), and any discrepancies were resolved through immediate discussion.

##### Group 1: Presence and Emotional Expectation

During the second round of evaluation, children were prompted to evaluate the two “good” and two “bad” paintings. E2 played an artist and came in with a painting with which the artist (an unfamiliar adult) was not satisfied. E1 asked E2 how their painting work was going. E2 expressed sadness, sighed, and said, “I worked really hard, but it seems that I am not good at it” E1 then said to the child, “Please evaluate this painting carefully and tell us your true opinion”

##### Group 2: Absence and Emotional Expectation

During the second round of evaluation, children were prompted to evaluate the two “good” and two “bad” paintings, after that, E1 took out a poor picture and asked the child what he/she thought of the picture, and said to the child, “This was painted by an artist, the artist worked really hard, but it seems that she was not good at it, she was very sad.” E1 then said to the child, “Please evaluate this painting carefully and tell us your true opinion”

##### Group 3: Presence and No Emotional Expectation

During the second round of evaluation, children were prompted to evaluate the two “good” and two “bad” paintings. E2 came in with their bad painting. E1 asked E2 how their painting work was going. E2, playing the role of an unfamiliar adult artist, expressed indifference and said, I’ve finished painting. I’m doing well and don’t care if I’m bad at drawing This wording was chosen to contrast with the emotional expectation condition by conveying a neutral attitude. After that, E1 reminded the child to talk to E2 for a minute.

##### Group 4: Absence and No Emotional Expectation

During the second round of evaluation, children were prompted to evaluate the two “good” and two “bad” paintings. E1 took out a poor painting and asked the child how it was. Additionally, E1 said to the child, “This painting was painted by an artist. The artist finally finished painting, but they doesn’t care about how it turned out”

### 2.3. Results and Discussion

The white lie rates broken down by condition and age groups are shown in Table 1. As indicated in the table, 75.3% of children in the emotional expectation and present condition were the most likely to tell white lies, compared with 17.2% of those who were the least likely to tell white lies in the no emotional expectation and absent condition. The χ^2^ test was used to further determine the difference between ages. The results showed that the condition difference was statistically significant (χ^2^ condition = 82.03, *p* < 0.001); 52.5% of primary school students were more likely to tell white lies than 39.4% of young children. The χ^2^ test was used to further determine the difference between ages. The results showed that the age difference was statistically significant (χ^2^ age = 6.53, *p* < 0.05).

We then conducted a binary logistic regression analysis with telling a white lie (0 = truth, 1 = white lie) as the predicted variable, and Age group (0 = primary school students, 1 = young children), Situational factor (1 = Emotional expectation and present, 2 = emotional expectation and absent, 3 = no emotional expectation and present, 4 = no emotional expectation and absent) and children type-by-situational factor as the predictor variables. EC was entered as the first step, children type and Situational factor as the second step, and their interaction as the final step. While the first step with EC was significant (B = 0.17, Wald = 7.71, *p* < 0.01); therefore, empathy was considered a control variable. The results in the second step showed that primary school students are more likely to tell white lies than young children, B = 0.57, Wald = 4.78, *p* < 0.05, and odds ratio [OR] = 1.76, which suggests primary school students who told white lies are 1.76 times higher than young children. Children in the emotional expectation and present were significantly more likely to tell the white lie than children in the no emotional expectation and absent condition, B = 2.76, Wald = 57.72, *p* < 0.001, and OR = 15.57, which suggests that the odds of children in the emotional expectation and present condition telling a white lie are 15.57 times higher than for those in the no emotional expectation and absent condition. Children in the emotional expectation and absent were significantly more likely to tell the white lie than children in the no emotional expectation and absent condition, B = 2.09, Wald = 36.30, *p* < 0.001, and OR = 8.05, which suggests that the odds of children in the emotional expectation and absent condition telling a white lie are 8.05 times higher than for those in the no emotional expectation and absent condition. Children in the no emotional expectation and present were significantly more likely to tell the white lie than children in the no emotional expectation and absent condition, B = 0.98, Wald = 8.03, *p* < 0.01, and OR = 2.66, which suggests that the odds of children in the no emotional expectation and present condition telling a white lie are 2.66 times higher than for those in the no emotional expectation and absent condition. There was a significant interaction between children’s age group and situational factors (B = 1.74, Wald = 5.41, *p* < 0.05), specifically, the effect of emotional expectation and presence on white lie behavior was more pronounced among primary school students (9–11 years old) than among younger children (4–5 years old). Further χ^2^ test found that the difference between white lie telling and truth telling was significant in the four experimental conditions (χ^2^ = 14.99, *p* < 0.01). Moreover, there was a significant difference between white lie telling and truth-telling among children of different ages under experimental conditions of emotional expectation (χ^2^ = 10.12, *p* < 0.01). The complete model of Study 1 is shown in Table 2.

Thus, Study 1 shows that when others had emotional expectations about their work and were present, children were most likely to tell white lies. Specifically, 86.4% of 9–11-year-olds, 71.7% of 6–8-year-olds, and 57.9% of 4–5-year-olds chose to tell white lies in the emotional expectation and present condition, indicating a gradual increase in white lie behavior with age and significant developmental differences. As children consider that an honest opinion may hurt another’s feelings or place them in an embarrassing situation, they tell white lies, reflecting their prosocial and people-oriented motivation. As children advance in age and attain more sophisticated levels of theory of mind, executive function, and empathy, the likelihood of white lying escalates (Sun et al., 2022a; Lv & Sun, 2022).

## 3. Study 2

People-oriented prosocial motivation is predominantly driven by empathy (Hudson & Jacques, 2014; Klimecki et al., 2016; Kline et al., 2019). Many studies have shown that empathy is the driving force of the dynamic system of prosocial behaviors such as sharing and helping others (Braun & Oertzen, 2021; Decety et al., 2018; Guo & Wu, 2021). When people experience empathy, they focus on the goals and needs of others (Horberg et al., 2011). White lies may not be their best choice when children consider the interests and goals of other people. However, so far, it is not clear what choices children will make when faced with conflicting prosocial values, causing a moral dilemma. When others have both emotional and target expectations, leading to a conflict between two prosocial inductions, will children make more rational choices and express honest opinions? The answer to this question is significant in helping us to understand the impact of moral conflict on children’s prosocial behavior. We created a situation where an adult “artist” brought their work to participate in a competition and sought the children’s opinions. The participants were primary scholars with high judgment ability and thinking development levels. Since the simple black-and-white strokes provided in Study 1 could not match the cognitive judgment of primary school students about adult entries, the experimental materials were upgraded to color gouache paintings. The settings of “good” and “bad” paintings in Study 1 were retained. The evaluation criteria of Study 1 were retained, but the item “whether the coloring is uniform and reasonable” was added to distinguish “good” from “bad” paintings.

Due to the limitations of preschoolers’ experience, intelligence levels, thinking development, weak emotional depth, stability, and self-control, it is difficult for them to understand the different benefits brought to others by emotional and goal cues (Heyman et al., 2020; Talwar et al., 2017). School-age children demonstrated improved cognitive ability and rational thinking. Low-grade primary school students are prone to their emotions and excitement. However, with the increase in age, their emotions are gradually internalized, and their emotional stability, balance, and profundity increase (Lupoli et al., 2017; Popliger et al., 2011). Their emotional reasoning is related to specific and objective things, and their thinking gradually transitions from thinking in terms of concrete images to abstract logic (Biccard & Wessels, 2011). During this stage, children can consider things in the long term and consider problems from the principle of value maximization (Sommerville et al., 2018). Therefore, primary school students were selected as the participants for Study 2. As in Study 1, video recording all sessions was not practical due to the sample size. Prior to the formal experiment, a pre-test was conducted with 30 lower-grade primary school children (as described in Section “Group 1: Presence and Target Expectation”). These sessions were video-recorded and independently coded by two experimenters. Inter-rater reliability was excellent (k = 0.91). During the formal experiment, two experimenters independently recorded children’s responses in real time, and any disagreements were resolved through discussion.

### 3.1. Participants

A total of 240 children between 6 and 11 years of age (Mage = 102.36 months, SD = 1.71, 50.4% boys) participated in the study. A priori power analysis (G Power 3.1; see Heyman et al., 2020) was run to determine the appropriate sample size for Study 2, which showed that a sample of 240 participants was sufficient to achieve 90% power. Participants were randomly assigned to one of two conditions, with 60 in each condition. Four of them were excluded due to a false distinction between “good” and “bad” in the first round of the task. The final sample of 236 children (Mage = 102.72 months, SD = 1.71, 49.6% boys). All of the remaining participants were predominantly from two primary schools in a large Eastern Chinese city. Using the same stratified random sampling method as Study 1, participants were stratified by grade and gender to ensure balanced age group representation, with sample size determined by power analysis. The study was approved by the research ethics committee in the Academy of Psychology and Behavior at Tianjin Normal University, China. All participants gave written informed consent before being enrolled in the experiment, which was obtained from their parents or legal guardians prior to the beginning of the study.

A test of empathy in children was the same as in Study 1. The task paradigm of painting evaluation was adopted in this study (Fu & Lee, 2007; Warneken & Orlins, 2015). Participants were randomly assigned to one of four conditions: Target expectation and present, target expectation and absent, no target expectation and present, and no target expectation and absent. Target expectation: The artist explicitly stated the goal of “participating in a competition and hoping to win” while expressing uncertainty about the work (“I worked really hard, but it seems I’m not good at it”), creating a conflict between emotional needs and goal needs. No target expectation: The artist only stated “I’ve finished painting for the competition” without emotional expression or goal emphasis, maintaining a neutral attitude.

### 3.2. Materials and Methods

#### 3.2.1. Empathy Test

The empathy test in Study 2 was the same as for Study 1.

#### 3.2.2. Test Procedure

**Figure 2:**
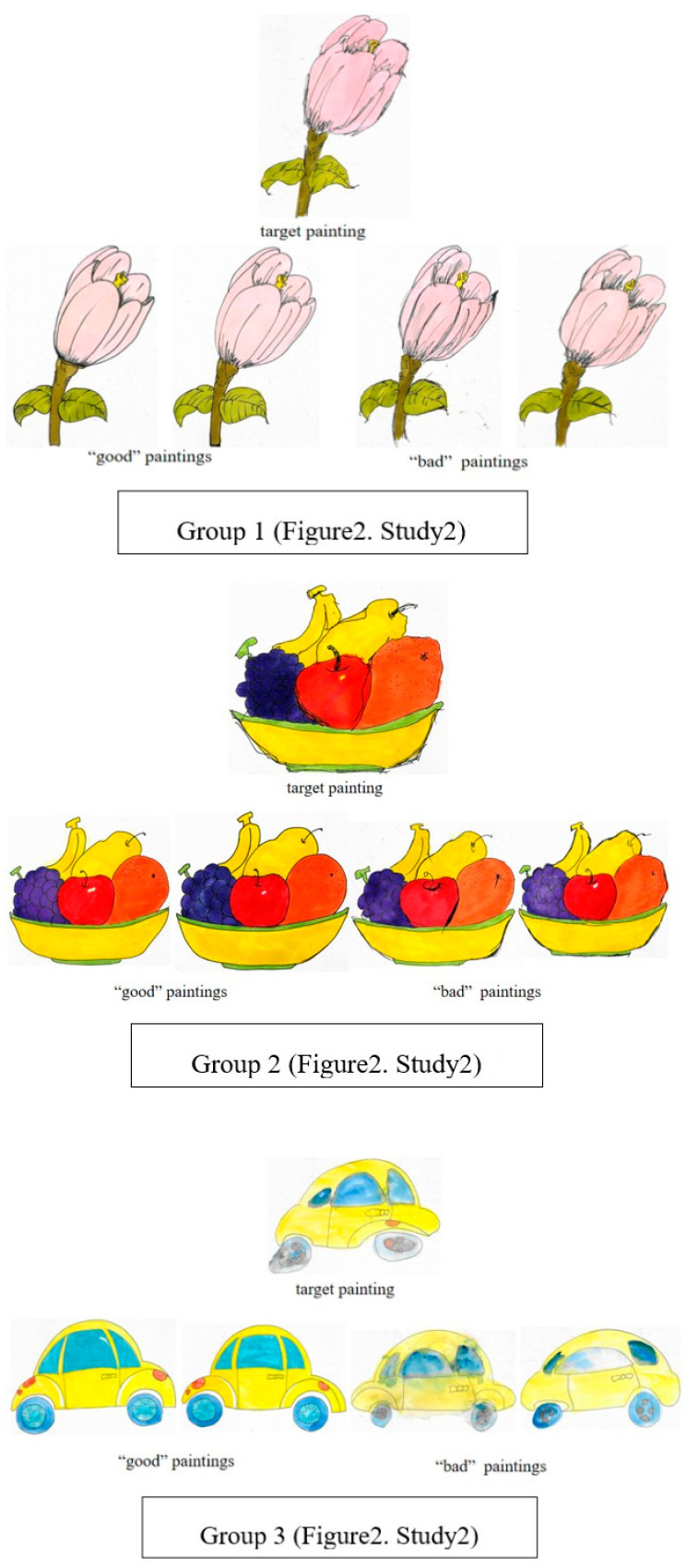
Example image of sample stimuli (Study 2).

Following the same theoretical framework as Study 1, we manipulated two types of motivational cues in Study 2. The emotional expectation cue was identical to that of Study 1, conveying sadness and desire for positive evaluation. The target expectation cue was introduced by having the artist explicitly state their goal of participating in a competition and hoping to win, while simultaneously expressing uncertainty about their work. This created a conflict between two prosocial motivations: protecting the artist’s feelings versus helping them improve for future success. The selection of these cues was guided by prior work on cost benefit analysis in children’s moral reasoning (Kool et al., 2010; Talwar et al., 2017), which suggests that children’s prosocial decisions involve weighing multiple, sometimes conflicting, social values. The experimental materials were three groups of color gouache paintings (flowers, fruit baskets, and cars; Figure 2), upgraded to match the cognitive level of primary school students and enhance task engagement. Each group contained five paintings: two “good” paintings with well-coordinated composition, uniform coloring, and smooth strokes; and three “bad” paintings with poor composition coordination, uneven coloring, and unsmooth strokes. Additionally, to ensure children perceived the artist’s effort, the target “bad” painting was presented with visual cues (e.g., sketches with revision traces placed next to it) indicating repeated attempts to improve. Notably, the core distinguishing criteria for “good” and “bad” paintings remained consistent with Study 1 to ensure cross-experimental comparability.

##### Group 1: Presence and Target Expectation

During the second round of evaluation, children were prompted to evaluate the two “good” and two “bad” paintings. E2 played an artist (an unfamiliar adult) and came in with a painting with which he was not satisfied. E1 asked E2 how his painting work was going. E2 expressed his sadness, sighed, said, “Ah, my painting is finally finished. I’m going to participate in the painting competition. I worked really hard, but it seems that I am not good at it” E1 addressed the participants, “This artist is going to enter a painting competition and hopes to make the painting better. If you say the painting is not good, the artist may feel sad but can revise it before the competition; if you say it’s good, the artist will be happy but may not make any changes. Please evaluate this painting carefully and tell us your true opinion” A pre-test was conducted with 30 lower-grade primary school students (6–7 years old) from the same schools but not participating in the formal experiment. They were asked to retell the explanation after hearing it, and the results confirmed that over 90% could accurately recap the core logic. The participant who told the truth (i.e., made negative comments) was further asked, (a) “Do you think that the painting is really bad?” and (b) “Do you want the artist to participate in the competition after modifying their painting?”. Answer (b) was deemed honest, and 0 points were given. If (a) was selected, the result was invalid.

##### Group 2: Absence and Target Expectation

During the second round of evaluation, children were prompted to evaluate the two “good” and two “bad” paintings. After that, E1 took out a poor picture and asked the child what they thought of the picture, and said to the child, “This painting was made by an artist who worked really hard but feels sad because it’s not good. The artist will enter a painting competition soon and hopes to make the painting better. If you say it’s not good, the artist may feel sad but can revise it; if you say it’s good, the artist will be happy but may not change it. The artist asked for your true evaluation. A pre-test confirmed that over 90% of lower-grade primary school students could retell the core logic of this explanation.

##### Group 3: Presence and No Target Expectation

During the second round of evaluation, children were prompted to evaluate the two “good” and two “bad” paintings. E2 (an unfamiliar adult) came in with their “bad” painting. E1 asked E2 how their painting work was going. E2 expressed indifference and said, “I’ve finally finished painting. I’m going to participate in the painting competition tomorrow, but I don’t care how the painting turned out or what the competition result will be” After that, E1 asked the child to evaluate E2’s painting.

##### Group 4: Absence and No Target Expectation

During the second round of evaluation, children were prompted to evaluate the two “good” and two “bad” paintings. E1 took out a poor painting and asked the child how it was. Additionally, E1 said to the child, “This painting was painted by an artist. The artist finally finished painting, but she doesn’t care about how it turned out. Please evaluate this painting carefully and tell us your true opinion”.

### 3.3. Results and Discussion

The white lie rates broken down by condition and age groups are shown in Table 3. As indicated in the table, 34.5% of children in the no target expectation and present condition were the most likely to tell white lies, compared with 11.9% who were the least likely to tell white lies in the target expectation and absent condition. The χ^2^ test was used to further determine the difference between ages. The results showed that the condition difference was statistically significant (χ^2^ condition = 9.98, *p* < 0.05). Moreover, 31.2% of 6–7-year-olds were more likely to tell white lies than 13.8% of 10–11-year-olds, and the χ^2^ test confirmed that this age difference was statistically significant (χ^2^ = 6.95, df = 2, *p* < 0.05), indicating a decreasing trend in white lie behavior as age increases in the target expectation scenario.

We then conducted a binary logistic regression analysis with telling a white lie (0 = truth, 1 = white lie) as the predicted variable, and age, situational factor (1 = Target expectation and present, 2 = target expectation and absent, 3 = no target expectation and present, 4 = no target expectation and absent) and age-by-situational factor as the predictor variables. EC was entered as the first step, age and situational factor as the second step, and their interaction as the final step. While the first step with EC was not significant. The results in the second step showed that children in the no target expectation and present condition are more likely to tell white lies than children in the no target expectation and absent condition, B = 0.95, Wald = 4.38, *p* < 0.05, and OR = 2.58, which suggests children in no target expectation and present condition who told white lies are 2.58 times higher than those in no target expectation and absent condition. Age-by-no target expectation and present term were significant (B = 0.84, Wald = 5.06, *p* < 0.05). Furthermore, the χ^2^ test found that there were no significant differences between white lies and truth-telling among children of different ages in the no target expectation and present condition. The complete model of Study 2 is shown in Table 4. In Table 4, Model 3 shows large standard errors for some interaction terms due to multicollinearity and limited cell sizes after stratification—parameter estimation issues, rather than computational errors. Thus, we interpret the more stable Model 2 as the primary analytic model; its results robustly support our conclusions. Future studies with larger samples should further examine higher-order interactions.

## 4. General Discussion

### 4.1. The Triggering Role of Situational Factors and Face Culture in White Lies

The present study explored the prosocial motivation behind children’s white lie behavior, guided by three hypotheses. Consistent with H1, children were more likely to tell white lies when the recipient was present, confirming the influence of face culture on children’s social decisions. H2 was also supported: emotional expectation significantly increased white lie rates, particularly in older children. H3 was partially supported: older children engaged in more cost benefit analysis and were more likely to tell the truth when it served the recipient’s long-term goals, especially in the absence of the recipient.

There are several important findings. Firstly, significant age differences were observed in children’s white lie behavior, which aligns with Piaget’s cognitive development theory. Younger children (4–5 years old, pre-operational stage) showed relatively rigid moral judgments and lower white lie rates, while older children (9–11 years old, concrete operational stage) demonstrated stronger perspective-taking ability (Sun et al., 2022b; Lv & Sun, 2022) a deeper perception of others’ feelings, and more rational white lie decision-making based on maximizing others’ overall interests. Secondly, no matter whether the other person showed emotional or target expectations for their work, children would consider the situational factor of whether the other person was present when evaluating. Even if children would like to consider the overall target interests of the other person and wanted to evaluate the work objectively, to enable the “artist” to win the prize, they displayed concern in the presence of the other person. However, in the absence of the other person, children were willing to speak freely and express their true evaluation of the painting. This reveals that children were extremely sensitive to the social environment, regardless of their age. Thirdly, children do not blindly tell white lies. Other people-oriented prosocial motivation would guide children to make decisions in the interests of others. If a white lie could comfort a sad person, children would choose to tell it. Nevertheless, after understanding that blindly telling white lies may not necessarily bring long-term benefits to the other person, children would do a cost benefit analysis for the other person and abandon lying rationally.

In Study 1, children’s empathy was induced by the other person’s emotional expectations for the painting, which enabled them to perceive the other person’s emotions. However, it is also possible that children responded to emotional cues more automatically, following a simple social script (“if someone is sad, be nice”) without engaging in deep empathetic processing. Future research should disentangle these two mechanisms by measuring children’s physiological responses or using implicit empathy tasks. In addition, children could predict that telling the truth might lead to negative emotions, while lying could probably arouse positive emotions in the other person. Therefore, they decided to tell a white lie. This finding verifies the previous Hypothesis 2. Previous studies have also supported this finding. Although children believe that lying is wrong, most children are willing to hide the truth when telling a white lie can spare another’s feelings or cheer them up (Ball et al., 2017; Harvey et al., 2018; Lupoli et al., 2017; Warneken & Orlins, 2015). The painting evaluation paradigm adopted in this study was designed to provide emotional cues (from the experimenter) to induce children to tell white lies for the sake of another. In this study, we investigated how children take the initiative to use prosocial rules to overcome social difficulties. Compared to young preschoolers, primary school students have an increasing ability to infer and distinguish the wishes and beliefs of others using emotional cues (such as facial expressions and body language). An additional theoretical lens that may illuminate our findings is the concept of salience. In social-cognitive decision-making, the relative prominence of competing cues can shift the weight children assign to different prosocial values. In Study 1, the artist’s physical presence combined with explicit emotional expressions (sadness and sighing) made the need for affective comfort highly salient, thereby biasing children toward white lies. When the artist was absent, these emotional cues became less salient, allowing children to focus more on the task’s objective evaluation criteria. This interpretation aligns with research showing that the salience of others’ emotional states directly influences children’s prosocial responses (Nagar et al., 2020; Kline et al., 2019). Meanwhile, increasingly, studies have shown that children are more likely to lie in the presence than the absence of the other person (Dvash & Shamay-Tsoory, 2014; Gallant et al., 2020; Shamay-Tsoory et al., 2010; Zanette et al., 2016). This is probably because, in some cases, children are required to abide by politeness rules and are taught by parents to display friendliness to their partners in interpersonal communication, especially when the other person is present. Fu and Lee (2007) believe that the criterion of flattery requires us to conceal negative feelings and views about another person when communicating face-to-face. In interpersonal communication, impression management is a foundational aspect of social life. During socialization, children learn to form their expected impressions through positive evaluations of others in social situations.

### 4.2. Developmental Shift Under Prosocial Value Conflict: From Empathy to Rationality

In Study 2, the experimenter created two kinds of social expectations, leading the children to face a conflict between two prosocial values. The results show that prosocial motivation guided children to do anything that could bring the greatest benefits to others, while paying more attention to the others’ goal of pursuing success than alleviating negative emotions. This finding can be understood through the concept of “social performance orientation” which refers to children’s tendency to prioritize socially valued outcomes (e.g., winning a competition) over immediate emotional comfort. This orientation was particularly evident in older children (9–11 years), who were more likely to tell the truth when it helped the artist improve their work and succeed in the competition, despite recognizing that honesty might cause temporary emotional distress. The age-related increase in social performance orientation suggests that with development, children internalize societal expectations regarding achievement and use them as benchmarks for prosocial decision-making, even when these conflict with politeness norms. However, this rational calculation was still moderated by face concerns when the recipient was present, indicating that social performance orientation operates within, rather than replaces, the cultural framework of face.

Older children demonstrated more rational white lie decision-making behavior and were sensitive to the presence or absence of the other person. The salience framework also helps explain the more complex patterns observed in Study 2. When the artist explicitly stated their goal of participating in a competition and hoping to win, the long-term benefits of honest feedback became more salient, particularly for older children who have internalized societal achievement norms. This heightened salience of future success may have tipped the cost benefit analysis toward truth-telling, despite the potential for immediate emotional harm. However, even when long-term goals were salient, the artist’s continued presence maintained the salience of face-related social norms, preventing a complete shift toward honesty. These findings suggest that children’s moral decisions in prosocial dilemmas are dynamically shaped by which aspects of a social situation are most prominent at the moment of decision.

### 4.3. Cost Benefit Analysis: The Rational Basis of Children’s Prosocial Decisions

This conclusion supports the previous research, Hypothesis 1, and Hypothesis 3. There is evidence that an increase in age leads to more people-oriented prosocial motivation in children. Their thinking changes from sensibility to rationality. Furthermore, their sense of rationality develops, and their emotions become more stable and mature. Some scholars have proposed cost benefit analysis, which guides individuals to make decisions, take actions, and form the foundation for inference and evaluation. Therefore, if the long-term benefits of a truth that seems harmful outweigh the temporary pain caused by telling the truth, children will choose to tell the truth. Although the choice of truthful evaluation will not meet the other person’s emotional expectations and might even cause sadness and depression for the other person, it can better meet the other person’s target expectations. This is because truthful evaluation can help the “artist” improve their painting skills and finally win the prize, which is their target expectation. Failing to meet others’ emotional expectations is judged as a small cost. Hence, a white lie is likely to lose long-term value, and this consequence is far greater than the short-term pain caused by telling the truth. Considering that children will rationally choose not to tell a white lie, older children are more likely to choose the option that can bring the greatest benefits, and are therefore more likely to tell the truth. However, when the other person is present, the truth may cause them to lose face and create a tense, embarrassing interpersonal mood. Children will try to avoid this negative interpersonal interaction. Hence, if the purpose of the white lie is to maximize the overall target interests of the other person, children are more willing to tell the truth in the absence than in the presence of the other person. The differential role of empathy across studies may reflect task demands. In Study 1, emotional cues directly triggered empathetic responses, making empathy a significant predictor. In Study 2, the presence of a moral dilemma may have activated cognitive cost benefit analysis, overriding the influence of empathy. This aligns with research showing that cognitive factors often dominate in complex moral decisions (Kool et al., 2010).

Collectively, these findings underscore the role of cue salience in shaping children’s prosocial decisions. When affective cues are salient (Study 1), immediate emotional considerations tend to dominate, biasing children toward white lies. Conversely, when long-term goals are rendered salient through explicit articulation (Study 2), older children increasingly prioritize future-oriented benefits—though concerns, activated by the recipient’s presence, remain sufficiently salient to moderate this rational calculation.

### 4.4. Limitations and Future Directions

Although we have obtained meaningful findings, this study has some limitations. Children’s empathy was taken as the control variable, but white lie behavior may also be affected by the participant’s personality traits, moral evaluation, social perception of goals, and family values. Future research can explore the mediating and regulating variables affecting children’s white lie behavior to reveal the influencing mechanisms more comprehensively. In addition, in this study, children were unfamiliar with the “artist” who is an adult, and there was an age difference between the children and the “artist.” The adult’s authority is a possible interference variable in this study. If the “artist” was a peer of the children, their evaluation would likely change. Furthermore, there are close and distant peer relationships. When evaluating a familiar friend, the familiar peer relationship would likely make children less rational. This is a potential direction for follow-up research. Additionally, the artist in this study was an unfamiliar adult, and future research could supplement a peer artist scenario to explore how familiarity with the target influences children’s moral decision-making. Thirdly, our task required children to physically place paintings into “good” or “bad” bags rather than verbally stating, “This is a good painting” While this behavioral measure reduces social pressure, it may differ from real-world situations where children must explicitly lie. Future studies could compare behavioral and verbal white lie measures to assess their equivalence. Fourthly, the wording in the no emotional expectation condition (“I don’t care”) may have introduced unintended connotations. Children might have perceived the artist as arrogant or insincere, potentially influencing their responses. Future research could systematically vary the phrasing of neutral emotional cues (e.g., “I’m just practicing” vs. “I don’t care”) to examine how subtle differences in wording affect children’s prosocial lying decisions. Although our design used unfamiliar adult experimenters and no explicit rewards or punishments to minimize authority effects, we acknowledge that children’s white lies may still reflect politeness toward an adult or concern about social disapproval. Future research could further disentangle these motivations by varying the artist’s age (peer vs. adult) or introducing explicit social consequences.

In this study, we found that when children were faced with a prosocial value conflict, they were inclined to treat the other person’s target expectation as the best solution for their overall interests. However, it is still unclear whether the children made the opposite choice because of incorrect predictions of others’ preferences and emotions or because they considered that the short-term goal was an urgent problem that needed to be solved. The answer to this question provides a direction for us to further understand empathy. Furthermore, future research could explore the factors affecting children’s value judgment of short-term and long-term goals. Most children believe that winning a prize in a painting competition may be the most beneficial choice for others. In other words, in most children’s values, hurting others’ feelings is inevitable in the process of striving for success. This predictive preference for other people’s values reflects their preference for values or is related to the children’s motivation to win. It is also unclear whether cultural differences exist in this phenomenon. For example, in China, competition has become a psychological incentive strategy reflecting self-worth and ability, frequently occurring in primary school students’ daily learning and living environment. The findings add to growing evidence of interconnections among culture, social cognition, and children’s prosocial behavior (Lim et al., 2020; Mojdehi et al., 2020). Further cross-cultural research can be conducted in the future. Future research could systematically manipulate cue salience by varying emotional intensity (e.g., mild disappointment vs. intense sadness) or goal explicitness (e.g., vague hopes vs. specific deadlines). Such manipulations would directly test whether the salience of affective versus future-oriented cues predicts children’s white lie decisions, providing more direct evidence for the role of cue salience in moral reasoning.

## 5. Conclusions

1. No matter whether the other person showed emotional or target expectations for their work, children would consider the situational factor of whether the other person was present when evaluating their work.
2. Children do not blindly tell white lies.
3. When others had emotional expectations about their work and were present, children were most likely to tell white lies.
4. Significant age differences were observed in children’s white lie behavior.
5. The consistency of instruction structure across experimental conditions, verified through post hoc analyses, supports the internal validity of these findings.

## Figures and Tables

**Table 1:** Percentage of children classified as white lie tellers, by age group and condition (N = 393) (Study 1).

	Young Children			Primary School Students			Total		
	*n*	White Lie-Teller	*Mage (SD)*	*n*	White Lie-Teller	*Mage (SD)*	*n*	White Lie-Teller	*Mage (SD)*
Emotional expectation and Present	38	57.9%	60.36 (0.91)	59	86.4%	106.32 (1.78)	97	75.3%	88.32 (2.40)
Emotional expectation and Absent	39	48.7%	55.44 (0.75)	60	71.7%	110.64 (1.46)	99	62.6%	88.8 (2.57)
No emotional expectation and Present	39	33.3%	57.24 (0.84)	59	35.6%	104.76 (1.87)	98	34.7%	85.8 (2.48)
No emotional expectation and Absent	39	17.9%	60.6 (0.89)	60	16.1%	110.64 (1.49)	99	17.2%	90.96 (2.41)

**Table 2:** Results of binary logistic regression on children’s white behavior (Study 1).

	Model 1 df = 1			Model 2 df = 4			Model 3 df = 8		
Variable	B	SE	OR	B	SE	OR	B	SE	OR
Constant	−1.15	0.39	0.32 **	−2.38	0.50	0.09	−2.05	0.57	0.13 ***
**EC**	0.17	0.06	1.19 **	0.09	0.08	1.10	0.11	0.08	1.11
**Children type**									
Young children				reference			reference		
Primary school students				0.56	0.26	1.74 *	−0.28	0.56	0.76
**Affective expectation type**									
Emotional expectation				1.88	0.24	6.53 ***	1.39	0.53	4.01 **
No emotional expectation				reference					
**Situational factor**									
Present				0.85	0.24	2.35 ***	0.82	0.54	2.28
Absent				reference					
**Children type × Affective expectation type**									
Primary school students × Emotional expectation							1.03	0.69	2.81
**Children type × Situational factor**									
Primary school students × Present							0.31	0.69	1.37
**Affective expectation type × Situational factor**									
Emotional expectation × Present							−0.44	0.71	0.64
**Children type × Affective expectation type × Situational factor**									
Primary school students × Emotional expectation × Present							0.40	0.97	1.49
−2 Log likelihood	535.78			449.83			442.07		
Cox & Snell *R*^2^	0.020			0.212			0.228		
Nagelkerke *R*^2^	0.027			0.284			0.304		

* *p* < 0.05; ** *p* < 0.01; *** *p* < 0.001.

**Table 3:** Percentage of children classified as white lie tellers, by age group and condition (N = 236) (Study 2).

	*Grade 1*			*Grade 2*			*Grade 3*			Total		
	*n*	White Lie-Teller	*Mage* *(SD)*	*n*	White Lie-Teller	*Mage* *(SD)*	*n*	White Lie-Teller	*Mage* *(SD)*	*n*	White Lie-Teller	*Mage* *(SD)*
Target expectation and Present	20	30.0%	79.20 (0.50)	20	30.0%	111.00 (0.44)	20	15.0%	103.20 (0.37)	60	25.0%	106.80 (1.82)
Target expectation and Absent	19	31.6%	81.48 (0.42)	20	5.0%	112.20 (0.49)	20	0.0%	129.00 (0.44)	59	11.9%	108.00 (1.70)
No target expectation and Present	19	36.8%	77.04 (0.51)	19	26.3%	104.16 (0.48)	20	40.0%	127.20 (0.50)	58	34.5%	103.20 (1.80)
No target expectation and Absent	19	26.3%	80.16 (0.49)	20	25.0%	102.60 (0.51)	20	0.0%	127.80 (0.49)	59	16.9%	103.92 (1.70)

**Table 4:** Results of binary logistic regression on children’s white behavior (Study 2).

	Model 1 df = 1			Model 2 df = 7			Model 3 df = 12		
Variable	B	SE	OR	B	SE	OR	B	SE	OR
Constant	−0.16	0.81	0.85	−2.88	1.21	0.06 *	−21.47	9220.76	0.00
**EC**	−0.16	0.12	0.85	0.09	0.15	1.10	0.03	0.16	1.034
**Grade**									
Grade1				1.26	0.49	3.52 *	20.24	9220.76	614,646,749.86
Grade2				0.67	0.45	1.95	20.16	9220.76	571,666,787.79
Grade3				reference			reference		
**Target expectation type**									
Target expectation				−0.55	0.36	2.30	0.01	12,875.68	999,938,889.80
No target expectation				reference			reference		
**Situational factor**									
Present				0.95	0.34	2.59 **	20.72	9220.76	1.01
Absent				reference			reference		
**Grade × Target expectation type**									
Grade1 × Target expectation							0.21	12,875.68	1.24
Grade3 × Target expectation							−1.91	12,875.68	0.15
**Grade × Situational factor**									
Grade1 × Present							−20.21	9220.76	0.00
Grade3 × Present							−20.66	9220.76	0.00
**Target expectation type × Situational factor**									
Present × Target expectation							−1.26	12,875.68	0.03
**Grade × Target expectation type × Situational factor**									
Grade1 × Target expectation × Present							0.68	12,875.68	1.97
Grade3 × Target expectation × Present							3.29	12,875.68	26.71
−2 Log likelihood	230.95			231.51			216.59		
Cox & Snell *R*^2^	0.073			0.071			0.128		
Nagelkerke *R*^2^	0.112			0.109			0.196		

* *p* < 0.05; ** *p* < 0.01.

## Data Availability

The original data presented in the study are openly available in Mendeley Data Sun, Yunrui; Du, Zhijie; Cui, Jinhai (2026), “Data for: Face Culture and Prosocial Value Conflict: A Developmental Investigation of Children’s White Lie Decisions Between Emotional Comfort and Long-Term Goals”, Mendeley Data, V1, doi: 10.17632/kh36rtrpt9.1. Note that some core data were preliminarily reported in a Chinese monograph by the first author (Sun, 2026). The present study conducts further in-depth statistical analysis and theoretical discussion based on the original dataset, with novel findings regarding the moderating role of face culture and developmental shifts in children’s prosocial decision-making that have not been reported previously.
